# Introduction of AGPAT3 gene as a regulator of cisplatin resistance in A2780 ovarian endometrioid carcinoma cell line

**DOI:** 10.1371/journal.pone.0318740

**Published:** 2025-03-10

**Authors:** Hadi Alizadeh, Sana Kerachian, Sadegh Babashah, Bahram M. Soltani

**Affiliations:** Department of Molecular Genetics, Faculty of Biological Sciences, Tarbiat Modares University, Tehran, Iran; Al-Azhar University/King Khalid University, EGYPT

## Abstract

Ovarian cancer therapy remains a challenge for human health, partly due to chemotherapy resistance. Understanding the molecular mechanisms underlying this resistance is crucial. Therefore, to identify genes involved in cisplatin resistance in ovarian cancer, RNA-seq analysis of A2780cp (cisplatin-resistant) and A2780 (cisplatin-sensitive) cell lines was performed, revealing 1-acylglycerol-3-phosphate O-acyltransferase 3 (*AGPAT3*) as a differentially expressed candidate gene. First, MTT analysis confirmed the drug resistance of A2780cp and the sensitivity of A2780 cell lines. Subsequent reverse transcription quantitative polymerase chain reaction (RT-qPCR) and western blotting analyses revealed elevated AGPAT3 and mTOR expression in A2780cp cells compared with A2780 cells. Additionally, western blotting showed increased p-mTOR (phospho-mTOR)/mTOR and p-S6K (phospho-S6K)/S6K ratios in A2780cp cells. The overexpression of AGPAT3 in A2780 cells led to increased p-mTOR/mTOR and p-S6K/S6K ratios and increased IC50 values, as shown by RT-qPCR, western blotting, and MTT analysis. Conversely, shRNA-mediated downregulation of AGPAT3 resulted in reduced p-mTOR/mTOR and p-S6K/S6K ratios. At the cellular level, AGPAT3 overexpression in A2780 cells increased survival rates, decreased apoptosis, and caused G2/M cell cycle arrest under cisplatin treatment, as detected by apoptosis assay, and cell cycle flow cytometry analysis. Overall, we conclude that AGPAT3 is involved in cisplatin resistance in A2780cp cells and propose that targeting this gene or its enzymatic product could help overcome drug resistance.

## 1. Introduction

Ovarian cancer is the eighth most prevalent cancer among women, with annual incidence and mortality rates of 6.6 and 4.2 per 100,000, respectively [1]. Cisplatin, a platinum-based compound, is a frontline chemotherapy agent for various solid tumors, including ovarian cancer [2]. Its mechanism involves disrupting DNA synthesis and transcription by forming purine DNA adducts, leading to apoptosis [3]. Despite an initial response to the drug, the development of cisplatin resistance eventually becomes a major challenge for most patients [4]. Mechanisms of resistance include alterations in drug influx or efflux, drug inactivation through interactions with glutathione and metallothionein, enhanced DNA repair, and modifications in signaling pathways [5].

The mammalian target of rapamycin (mTOR) acts as a crucial regulator of cell growth, proliferation, and metabolism [6]. The mTOR protein forms two complexes, mTORC1 and mTORC2, each of which differ in composition, upstream and downstream contributors, and sensitivity to rapamycin. One of the cofactors of the mTOR signaling pathway is phosphatidic acid (PA), which primarily affects mTORC1 [7]. PA enhances mTORC1 kinase activity by directly binding to the FK506-binding protein 12 (FKBP12)-rapamycin-binding (FRB) domain of mTORC1 [8]. Overactivation of mTORC1 promotes the phosphorylation of downstream targets such as S6K1 (Ribosomal protein S6 kinase beta-1) and 4E-BP1 (eukaryotic translation initiation factor 4E-binding protein 1) [9]. PA is produced by three different enzymes: phospholipase D (PLD), diacylglycerol kinase (DGK), and 1-acylglycerol-3-phosphate O-acyltransferase 3 (AGPAT) or lysophosphatidic acid acyltransferase (LPAAT) [10]. Acyl glycerol phosphate acyltransferases (AGPATs) are enzymes that catalyze the conversion of lysophosphatidic acid to PA [11,12].

A recent study utilizing tissue microarrays revealed that mTORC1 activation is significantly higher in ovarian clear-cell carcinomas, with 86.6% of cases showing increased activation, compared to 50% of serous adenocarcinomas [13]. This heightened activation of mTORC1 plays a crucial role in enhancing cellular resistance to cisplatin through several mechanisms. These include facilitating G2/M checkpoint recovery following DNA damage [14], boosting DNA repair processes [15–18], inhibiting apoptosis, and promoting overall cell survival [19]. Furthermore, the central role of mTORC1 in cisplatin resistance has been reported in various cancers, including gastric, cervical, and lung cancers. Inhibition of mTORC1 has demonstrated potential to sensitize cancer cells to cisplatin, highlighting its promise as a therapeutic target to overcome chemoresistance [20–22]. Previous studies have associated AGPAT2 with mTOR regulation, demonstrating its role in cisplatin resistance [23]. In this study, we hypothesize that AGPAT3 activation contributes to increased mTORC1 activation and decreased cisplatin-induced apoptosis. To validate our hypothesis, we used A2780 cell line as a model for ovarian cancer established from an endometrioid-type ovarian tumor [24,25]. Understanding the role of AGPATs in cisplatin resistance may pave the way for novel mTOR inhibitors, enhancing the efficacy of cisplatin chemotherapy.

## 2. Materials and methods

### 2.1. RNA-seq analysis

For transcriptome profile analysis, public RNA-seq datasets (GSE173201, GSE176218, GSE98230, and GSE206649) taken from SRA database (https://www.ncbi.nlm.nih.gov/sra) containing A2780 and A2780cp samples were utilized. Data processing and analysis were conducted on the Galaxy platform (https://usegalaxy.org). Raw sequencing reads were subjected to quality control assessment via FastQC (0.74+galaxy0), followed by trimming of low-quality bases and adapter sequences via Trim Galore (0.6.7+galaxy0) and Trimmomatic (0.39+galaxy2). Alignment was performed with HISAT2 (2.2.1+galaxy1) with hg38 reference genome, and read counting was conducted via HTSeq-count (2.0.5+galaxy0). Batch effect removal was carried out via the Combat-seq tool from SVA package (3.46.0) in R. Normalization and differential gene expression analysis were performed via DESeq2 (version 1.38.3) R. Principal component analysis (PCA) was performed with DESeq2 to validate sample clustering and evaluate the overall datasets variability. Visualization of expression patterns and differentially expressed genes (DEGs) was facilitated through the generation of heatmaps (circlizing 0.4.15) and volcano plots (EnhancedVolcano 1.16.0).

### 2.2. Enrichment analysis

To gain insight into the biological significance of the DEGs, enrichment analysis was performed with Clusterprofiler (4.6.2) package, and visualization of enrichment result was performed with Enrichplot package, which uses Kyoto Encyclopedia of Genes and Genomes (KEGG) database for signaling pathway analysis. This analysis helps identify active pathways and processes linked to DEGs, improving our understanding of the biological mechanisms involved in cisplatin chemoresistance.

### 2.3. Cell culture and cisplatin treatment

A2780 and A2780 cisplatin-resistant (A2780cp) cell lines were obtained from Pasteur Institute (Tehran, Iran). Both cell lines were maintained in RPMI 1640 medium supplemented with 10% fetal bovine serum (FBS; Gibco, Brazil), 100 U/ml penicillin, and 100 µg/ml streptomycin. The cells were cultured in a humidified incubator at 37 °C with 5% CO2 and passaged according to standard protocols. A2780 cells were treated with 20 µM, 35 µM, 50 µM, 65 µM, and 80 µM cisplatin. Cisplatin was diluted in the cell culture medium to achieve the desired concentrations. The cells were incubated with cisplatin for 24 hours under standard culture conditions.

### 2.4. RNA extraction and cDNA synthesis

Total RNA was extracted from cultured cells via the TRIzol reagent of Zavarzistazma (Kerman, Iran). Subsequently, complementary DNA (cDNA) synthesis was performed via Reverse Transcription kits from Thermo Fisher (USA) and Yekta Tajhiz (Tehran, Iran) Azma. Both procedures were carried out following the manufacturers’ instructions. The quantity and quality of RNA were assessed via a NanoDrop spectrophotometer, and the integrity of RNA was confirmed via gel electrophoresis.

### 2.5. Reverse transcription-quantitative polymerase chain reaction (RT‒qPCR)

Gene expression levels were further assessed via RT-qPCR with SYBR Green detection. Quantitative real-time PCR was performed with SYBR Green (Amplicon, Denmark) and gene-specific primers (Table 1) for the target genes, with Actin Beta-1 (ACTB1) as an internal control. The expression levels were normalized to ACTB1 gene, and relative quantification was determined via the ∆∆Ct method. Each experiment was performed in two biological replicates (n = 2) with two technical repeats each (n = 2).

**Table 1:** Primers used in this study.

Primer	Forward	Reverse
ACTB	5′- TGGACTTCGAGCAAGAGATG-3′	5′ -GAAGGAAGGCTGGAAGAGTG-3′
AGPAT3 (Real-time)	5′- CCCGAGTACATGTGGTTTCTC-3′	5′ -GTCCTTCTCCTGGTACAGTTTATG-3′
AGPAT3(Cloning)	5′- GACGGCTGTCCTCAGCGAG-3′	5′ -TCTTTTCCTGCACTCTGTGTGTTG-3′
shRNA	5′-GGTGGATCCTCAACTGCCGCCTCG CCTACTTTGTAGAG-3′ (including BamHI site)	5′-GGTAAGCTTTCAACTGCCGCCTC GCCTACTCTACAAAG-3′ (including HindIII site)
mTOR	5′ -GCAGCAACAGTGAGAGTGAGG-3′	5′ -GACAAGGAGATGGAACGGAAGAA-3′

### 2.6. Gene cloning

The gene cloning process involved the amplification of AGPAT3 cDNA derived from A2780 cell line via PCR via gene-specific primers (Table 1) designed for coding sequence (CDS) of AGPAT3 gene. The PCR products were purified via the Favorgen (Tehran, Iran) Gel/PCR Purification Kit. First, TA-cloning was performed, and then the AGPAT3 sequence was digested with the restriction enzymes APA1 and KPN1 (Takara, Japan) from the recombinant T-vector. The purified AGPAT3 gene fragment was then ligated into the pcDNA3.1+ vector via T4 DNA ligase as part of a standard cloning protocol. The ligated recombinant vectors were subsequently transformed into competent DH5-Alpha bacteria via the heat shock method. Transformed bacteria were selected via LB agar ampicillin, and the presence of the recombinant AGPAT3 gene was confirmed via colony PCR. Sanger sequencing confirmed the accuracy of the cloned-AGPAT3 sequence.

### 2.7. Designing the shRNA

The CDS of AGPAT3 was submitted to the UNAFold web server (unafold.org) for mRNA structural analysis and folding to identify the optimal shRNA target position. Based on the analysis, the shRNA was designed with 2 primers (Table 1). PCR was used to anneal these primers to form a dimer and complete polymerization, creating the shRNA construct (S1 Fig).

### 2.8. Transient transfection

A2780 cells underwent transient transfection to establish mock and overexpressing cell lines. Transfection was performed via the Thermo Fisher Lipofectamine 3000 reagent (USA) according to the manufacturer’s instructions, and the pcDNA3.1(+) expression vector contained the target gene AGPAT3 and pRNA vector, which contained shRNA. For overexpression of the AGPAT3 gene in A2780 cells, the cells were seeded in the appropriate plates according to the methods of the downstream assay and incubated for 24 hours to allow adherence and reach approximately 70% confluency. For transfection, a mixture of Lipofectamine 3000, P3000, and recombinant vector pcDNA3.1(+):AGPAT3 was prepared in medium without FBS. The ratios used were as follows: for MTT assay, 5000 cells per well were seeded in 96-well plates, and 0.2 µL of Lipofectamine 3000, 0.3 µL of P3000, and 0.3 µg of DNA were used per well; for RT-qPCR, apoptosis, and cell cycle assays, 90000 cells per well were seeded in 12-well plates, and 2 µL of Lipofectamine 3000, 3 µL of P3000, and 3 µg of DNA were used per well; for Western blotting, 250000 cells per well were seeded in 6-well plates, and 4 µL of Lipofectamine 3000, 6 µL of P3000, and 6 µg of DNA were used per well. After the transfection mixture was added to the cells, the plates were incubated for 6 hours, after which the medium was replaced with fresh complete medium. For downregulation of AGPAT3 in A2780cis cells, the same protocol was followed for the recombinant pRNA:shRNA vector, and downregulation was confirmed by RT-qPCR and Western blotting.

### 2.9. MTT assay

Cell viability was assessed via the MTT assay. A2780 and A2780cp cells were seeded in 96-well plates. After the 24-h treatment period, MTT solution (5 mg/ml) was added, and the cells were incubated for 3–4 h at 37°C. Then, dimethyl sulfoxide (DMSO) was added, and the absorbance was measured at 570 nm via an ELISA reader. Cell viability was calculated by comparing the absorbance of treated and untreated (control) cells. The experiment was performed with three biological replicates (n =  3) with three technical repeats each (n =  3). The IC50 values were calculated with GraphPad Prism (version 9).

### 2.10. Western blot analysis

Harvested cells were lysed in lysis buffer, and protein concentrations were measured via the Bradford assay. Western blotting was performed to detect AGPAT3, mTOR, p-mTOR, S6K, and p-S6K proteins. For each sample, total proteins were separated via 10% sodium dodecyl sulfate‒polyacrylamide gel electrophoresis (SDS-PAGE) and transferred to a PVDF membrane. The membrane was blocked with 5% BSA in TBST buffer and then incubated overnight at 4°C with the following primary antibodies directed against AGPAT3 (rabbit, 1:1000;25723-1-AP, Proteintech), mTOR (mouse, 1:500; sc-517464, Santa Cruz Biotechnology, Inc.), p-mTOR (Ser 2448) (mouse, 1:500; sc-293133, Santa Cruz Biotechnology, Inc.), p70 S6 kinase α (rabbit, 1:500; sc-230, Santa Cruz Biotechnology, Inc.), p-p70 S6 kinase α (Thr 389) (rabbit, 1:500; sc-11759-R, Santa Cruz Biotechnology, Inc.), and β-actin (mouse, 1:2000; sc-517582, Santa Cruz Biotechnology, Inc.). After washing, the membrane was incubated with horseradish peroxidase (HRP)-conjugated secondary antibodies directed either against mouse IgG (m-IgGκ BP-HRP, 1:5000; sc-516102, Santa Cruz Biotechnology, Inc.) or against rabbit IgG (mouse, anti-rabbit IgG-HRP, 1:5000; sc-2357, Santa Cruz Biotechnology, Inc.). Protein bands were visualized via an enhanced chemiluminescence (ECL) kit (Lumigen, USA) according to the manufacturer’s instructions and imaged via a chemiluminescence imaging system.

### 2.11. Cell cycle analysis

Following AGPAT3 overexpression, A2780 cells were collected and stained with propidium iodide (PI). The distribution of cells in each phase (Sub-G1, G1, S, and G2/M) was detected via a BD FACS Calibur (BD Biosciences, San Jose, USA), and the results were analyzed with FlowJo V10 software.

### 2.12. Apoptosis assay

After AGPAT3 was overexpressed in A2780 cells, the cells were collected and stained with an Annexin-V-FITC/PI Staining Kit. Next, a BD FACS Calibur (BD biosciences, San Jose, USA) was used to detect apoptotic and viable cell rates, and FlowJo V10 was used to analyze the results.

### 2.13. Statistical analysis

Statistical analyses were conducted using GraphPad Prism 9 software (GraphPad Software Inc., San Diego, CA, USA). Student’s t test was employed for the comparison of two normally distributed variables.

### 2.14. Ethics statement

This study did not involve human participants; therefore, obtaining participant consent was not applicable. The research did not include any personal data, medical records, or biological samples related to human subjects.

## 3. Results

### 3.1. Bioinformatic analysis revealed AGPAT3 as a differentially expressed gene between A2780 and A2780cp cell lines

**Fig 1:**
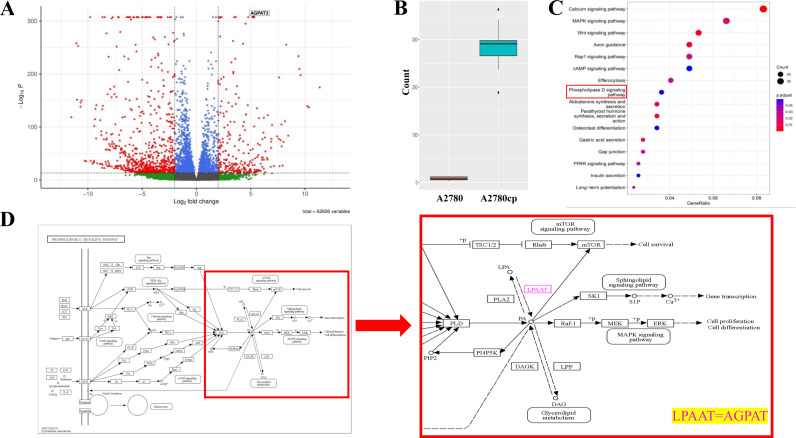
Introduction of potential genes and signal pathways related to cisplatin resistance in the A2780 cell line based on Bioinformatics analysis. **(A)** Volcano plot of the results of the RNA-sequencing analysis of differentially expressed genes in A2780 vs. A2780cp, with AGPAT3 highlighted. **(B)** Fold change of AGPAT3 gene expression from RNA-Seq data analysis between A2780 and A2780cp cells. **(C)** Illustration of the pathways involved in the cisplatin resistance phenotype, including phospholipase D signaling, as determined by enrichment analysis. **(D)** Part of phospholipase D signal pathway obtained from the KEGG database showing the interaction between phospholipase D and the mTOR signaling pathway (LPAAT, also known as AGPAT).

To characterize the expression profile of A2780cp, we conducted RNA-seq analysis utilizing SRP316044, SRP322864, SRP105266, and SRP383043 datasets retrieved from the NCBI. Batch effect removal procedure was applied to ensure the elimination of biases introduced by batches. A volcano plot (Fig 1A) and PCA (S2 Fig) confirmed the accuracy of the analysis. After removing genes with p-adj >  0.05 and | log2(FC) | >  1, we identified 1505 upregulated and 3347 downregulated DEGs (S1 File). *AGPAT3* had a base mean of 146.7 and a log2(FC) of 5.13 (Fig 1B). Further enrichment analysis identified phospholipase D as one of the potential signaling pathways associated with cisplatin chemoresistance in A2780 cells (Fig 1C). Seventeen upregulated genes, including *AGPAT3*, from the RNA-seq data belonged to the phospholipase D signaling pathway (S1 File). After a stringent threshold of base mean >  50 (S1 File), a heatmap was generated, and *AGPAT3* ranked 25^th^ among the upregulated genes (S3 Fig). Signal pathway analysis via the KEGG database revealed the interaction of phospholipase D with mTOR signaling pathway (Fig 1D).

### 3.2. AGPAT3 mRNA and protein levels were elevated in A2780cp cells compared with A2780 cells

**Fig 2:**
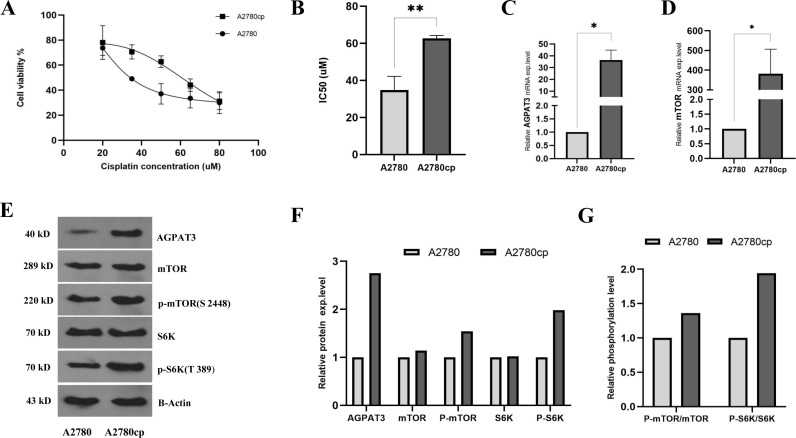
Characterization of cisplatin resistance phenotype in A2780 and A2780cp cells. **(A)** Cell survival comparison between A2780 and A2780cp cells was conducted using MTT assay with five different concentrations of cisplatin. The experiment included three biological replicates, each with three technical repeats. **(B)** Cisplatin IC50 values for A2780 and A2780cp cells were determined via the best-fit dose-response model in Graph Pad Prism. **(C)** RT-qPCR results indicating increased transcription level of the AGPAT3 gene in A2780cp cells compared to A2780 cells. **(D)** RT-qPCR results indicating increased transcription level of mTOR gene in A2780cp cells compared with A2780 cells. **(E)** Western blot analysis against protein levels of AGPAT3, mTOR, p-mTOR, S6K, and p-S6K in A2780cp and A2780 cells. **(F)** Quantification of the western blot analysis results verified elevated levels of AGPAT3, mTOR, p-mTOR, and p-S6K proteins in A2780cp cells compared with control cells. **(G)** Relative phosphorylation levels of mTOR and S6K in A2780 and A2780cp cells, indicating overactivation of the mTORC1 signaling pathway in A2780cp cells.

To verify the cisplatin resistance/sensitive phenotype of the cells, an MTT assay was performed on A2780 and A2780cp cell lines. The results revealed a greater number of viable A2780cp cells (drug-resistant phenotype) treated with different concentrations of cisplatin than sensitive A2780 cells (Fig 2A). Accordingly, IC50 values of 34.08 µM and 62.62 µM were deduced for A2780 and A2780cp cells, respectively (Fig 2B). Subsequently, RT-qPCR analysis revealed significant upregulation of AGPAT3 (Fig 2C) and mTOR transcripts (Fig 2D) in A2780cp cells compared with A2780 cells. Furthermore, western blot analysis confirmed the elevated protein level of AGPAT3 in A2780cp cells, along with higher phosphorylation levels of mTOR and S6K proteins compared to those in A2780 cells, indicating overactivation of the mTORC1 complex (Fig 2E–G).

### 3.3. AGPAT3 overexpression in A2780 cells overactivated mTORC1 signaling pathway

**Fig 3:**
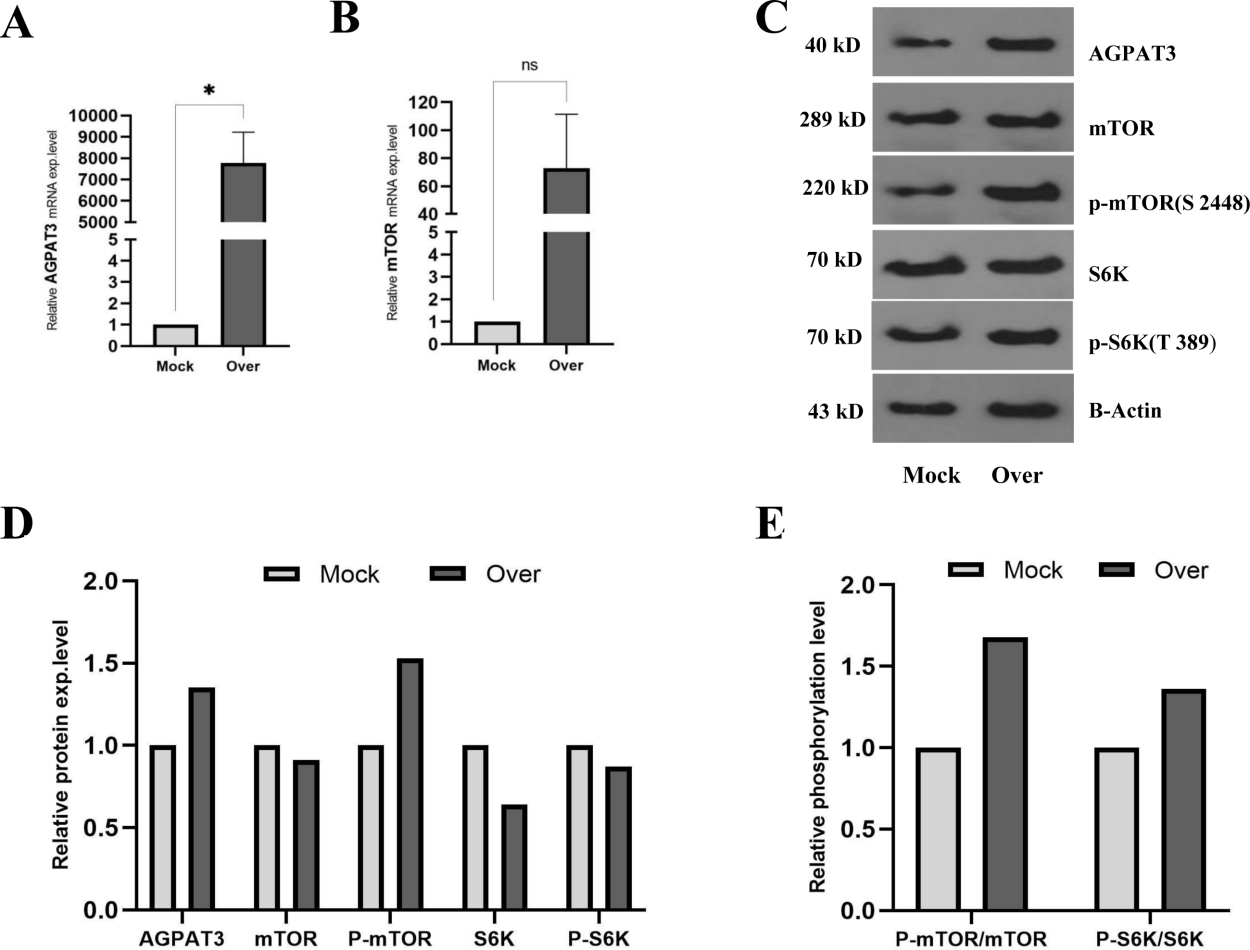
Effect of AGPAT3 overexpression on mTORC1 signaling pathway. **(A)** RT-qPCR analysis of AGPAT3 mRNA level in A2780 cells transfected with a recombinant vector confirming AGPAT3 overexpression compared with mock-transfected cells after DNase treatment. These results indicate successful AGPAT3 overexpression. **(B)** RT-qPCR analysis of mTOR mRNA level in A2780 cells following AGPAT3 overexpression compared with that in mock-transfected cells, showing elevated mTOR transcription level. **(C)** Western blot analysis of AGPAT3, mTOR, p-mTOR, S6K, and p-S6K protein levels following AGPAT3 overexpression in A2780 cells. β-actin was used as an internal and loading control. **(D)** Quantification of the western blot analysis results showing elevated protein levels of AGPAT3 and p-mTOR and reduced S6K and p-S6K protein levels in transfected cells following AGPAT3 overexpression. **(E)** Relative phosphorylation levels of mTOR and S6K in A2780 cells transfected with the mock or AGPAT3 overexpression vector, indicating overactivation of the mTORC1 signaling pathway in A2780 cells following AGPAT3 overexpression.

The successful overexpression of *AGPAT3* was confirmed through RT-qPCR against cDNA preparations of DNase-treated RNA samples obtained from transfected A2780 cells (Fig 3A). Additionally, RT-qPCR analysis revealed an increase in the mTOR transcription level in A2780 cells overexpressing *AGPAT3* (Fig 3B). Interestingly, western blot analysis (Fig 3C) revealed that, compared with those in mock-transfected cells, the protein levels of only *AGPAT3* and p-mTOR were elevated, whereas the protein levels of the other tested genes were decreased (Fig 3D). However, measuring the ratios of phosphorylated to nonphosphorylated protein levels of mTOR and S6K indicated an increase in the phosphorylated forms of both proteins (Fig 3E).

### 3.4. Attenuation of mTORC1 signaling pathway following the downregulation of AGPAT3 in A2780cp cells

**Fig 4:**
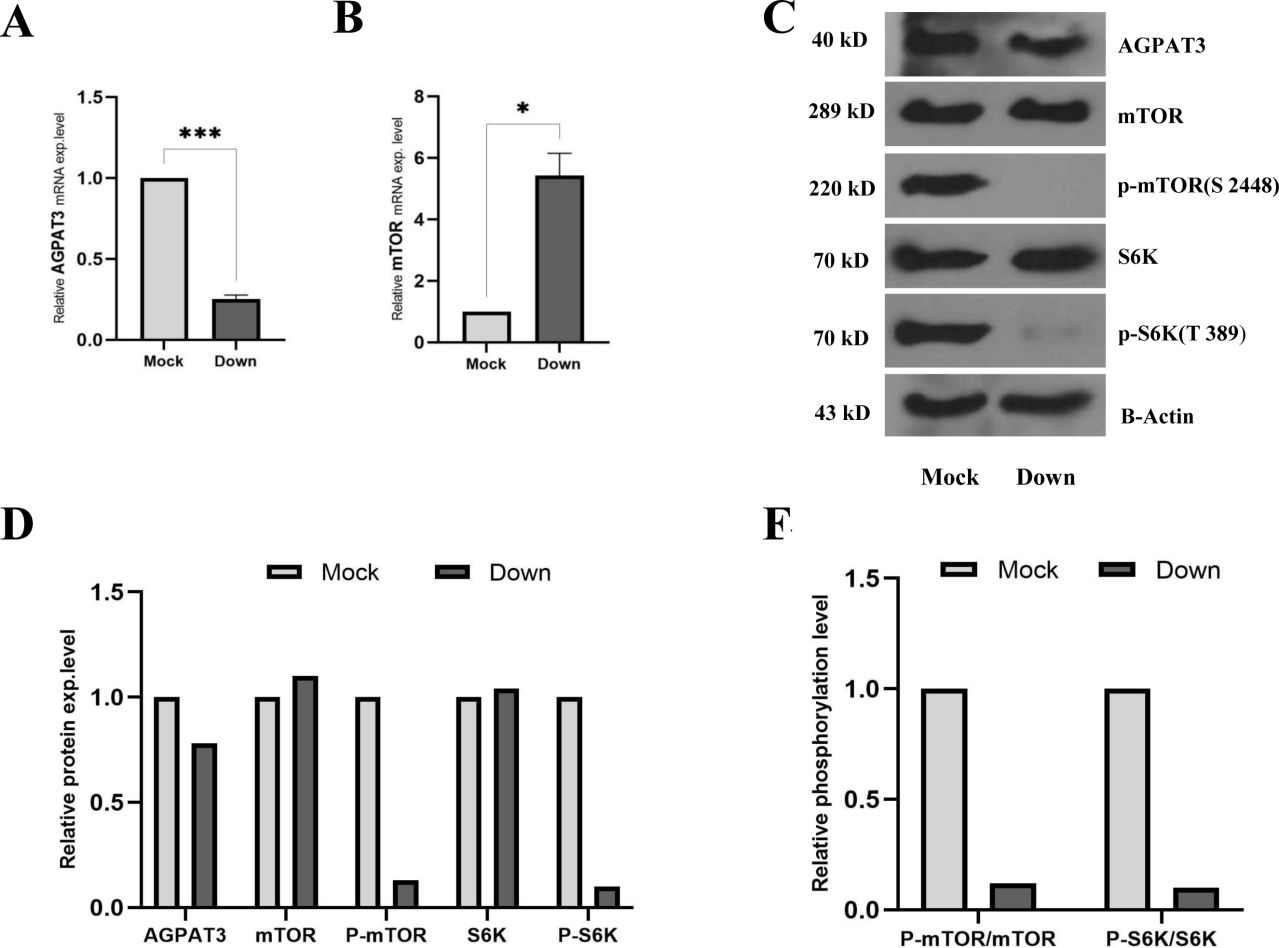
Effect of AGPAT3 downregulation on the mTORC1 signaling pathway. **(A)** RT-qPCR result showing successful downregulation of AGPAT3 using specific shRNA in A2780cp cells. **(B)** RT-qPCR result indicating upregulation of mTOR transcription level following AGPAT3 downregulation in A2780cp cells. **(C)** Western blot analysis of AGPAT3, mTOR, p-mTOR, S6K, and p-S6K protein levels following AGPAT3 downregulation in A2780cp cells. β-actin was used as an internal and loading control. **(D)** Quantification of the western blot analysis results showing a successful reduction in AGPAT3 protein levels, accompanied by reduced p-mTOR and p-S6K protein levels, in transfected A2780cp cells. **(E)** Relative phosphorylation levels of mTOR and S6K, indicating the attenuating effect of AGPAT3 knockdown on the mTORC1 signaling pathway.

To decrease the expression level of AGPAT3, a specific shRNA was cloned and inserted into pRNA vector and transfected into A2780cp cells. RT-qPCR analysis of AGPAT3 expression indicated approximately 75% successful downregulation of this gene compared with that in mock-transfected cells (Fig 4A). Surprisingly, RT-qPCR analysis showed an increase in mTOR mRNA levels under these circumstances (Fig 4B). Despite the increased mTOR protein level, western blot analysis verified that the p-mTOR protein level drastically decreased following the reduction in AGPAT3 protein levels (Fig 4C and D). Consequently, the level of the phosphorylated form of the S6K protein (p-S6K) also decreased under this condition (Fig 4C and D). This effect was more pronounced when the ratios of p-mTOR/mTOR and p-S6K/S6K were calculated (Fig 4E), demonstrating a strong correlation between AGPAT3 and the mTORC1 signaling pathway.

### 3.5. AGPAT3 overexpression was followed by cisplatin chemoresistance in A2780 cells

**Fig 5:**
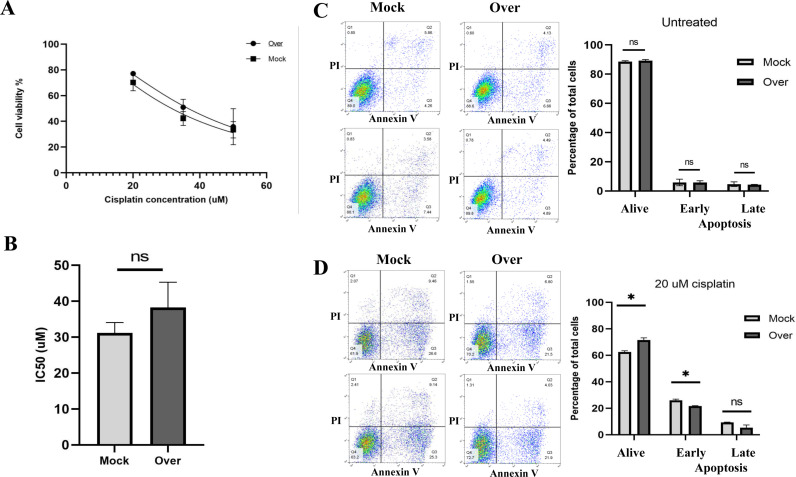
Viability and drug resistance of A2780 cells following AGPAT3 overexpression. **(A)** MTT assay results showing increased viability of A2780 cells overexpressing AGPAT3 compared with mock-transfected cells after treatment with three different concentrations of cisplatin (20, 35, and 50 µM). The experiment included two biological replicates and three technical repeats. **(B)** The graph indicates the increased IC50 of A2780 cells (from 31.2 to 38.21 µM) following AGPAT3 overexpression. **(C)** PI-annexin analysis results of A2780 cells overexpressing AGPAT3, indicating no significant effect on apoptosis in transfected cells without cisplatin treatment. **(D)** PI-annexin analysis results of A2780 cells overexpressing AGPAT3 under cisplatin treatment, showing a significant protective effect in transfected cells, as demonstrated by an increased number of live cells and reduced number of apoptotic cells.

To investigate the effect of AGPAT3 overexpression on the cisplatin resistance phenotype of A2780 cells, an MTT assay was performed. The cells were transfected with a vector ensuring AGPAT3 overexpression and then treated with different concentrations (20, 35, and 50 µM) of cisplatin after 24 hours. The MTT assay was conducted 24 hours after drug treatment. The results indicated that AGPAT3 overexpression led to an increased number of viable cells at all the tested drug concentrations (Fig 5A) and increased the IC50 value of A2780 cells from 31.2 to 38.21 µM (Fig 5B). To investigate the impact of AGPAT3 overexpression on the apoptosis rate of A2780 cells under both untreated and 20 µM cisplatin treatment conditions, a flow cytometry-based apoptosis assay was conducted. Under non-treatment conditions, no significant changes in cell survival and apoptosis were observed in the cells overexpressing AGPAT3 (Fig 5C). However, a significant increase in cell survival rate and a reduction in apoptosis rate were observed in the cells overexpressing AGPAT3, under cisplatin treatment (Fig 5D), confirming the role of AGPAT3 in conferring chemoresistance to cisplatin in A2780 cells.

### 3.6. Overexpression of AGPAT3 increased the proportion of A2780 cells in the G2/M phase under cisplatin treatment in A2780 cells

**Fig 6:**
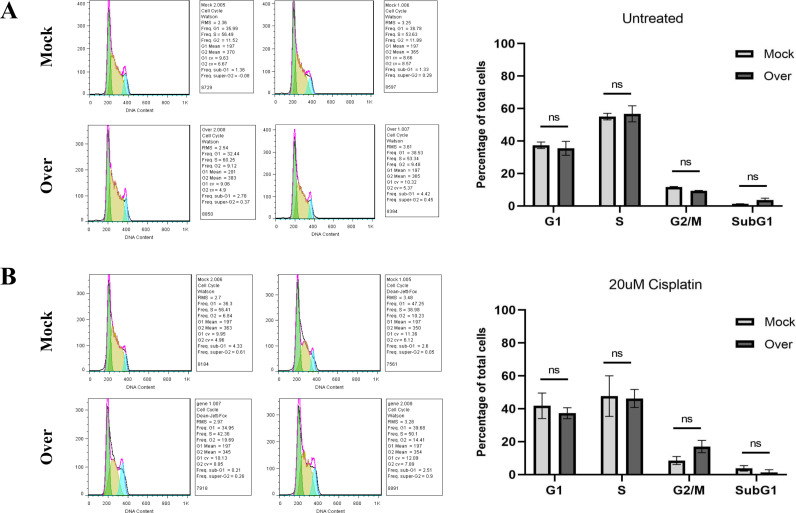
Cell cycle analysis of A2780 cells following AGPAT3 overexpression. **(A)** Cell cycle analysis of A2780 cells overexpressing AGPAT3, indicating no significant alteration in cell cycle distribution without cisplatin treatment. **(B)** Cell cycle analysis of A2780 cells overexpressing AGPAT3 revealed an elevated G2/M ratio following cisplatin treatment.

The cell cycle analysis revealed that there were no significant changes in the distribution of cell cycle phases in AGPAT3-overexpressing cells compared to the mock-transfected cells (Fig 6A). However, in the presence of 20 µM cisplatin, AGPAT3 overexpression resulted in a notable increase in the percentage of cells in the G2/M phase compared with that in the mock group (Fig 6B). Specifically, in the mock group, the percentage of cells in the G2/M phase was 8.53%, whereas in the AGPAT3-overexpressing group, it increased to 17.05%. These results further support the chemoresistance effect of AGPAT3 overexpression in these cells.

## 4. Discussion

Ovarian cancer ranks as one of the most prevalent malignancies of the gynecological system [1]. Although the majority of ovarian cancer patients achieve complete clinical remission, 50-70% of them experience tumor recurrence due to chemoresistance [2]. Therefore, elucidating the molecular mechanisms involved in the chemoresistance phenotype is crucial.

Here, we used RNA-seq and enrichment analysis to identify differentially expressed genes involved in the chemoresistance phenotype of A2780 cells, an ovarian cancer-derived cell line. *AGPAT3* was chosen for further analysis because of its relatively high base mean and log2(FC) of 5.13 between A2780cp and A2780 cells. Previously, the oncogenic role of *AGPAT2*, another member of the AGPAT gene family, has been reported in leukemia [26], pancreatic [27], and ovarian cancers [28–30], as well as in cisplatin chemoresistance in osteosarcoma [23]. Based on these findings, we hypothesized that *AGPAT3* might play a similar role in cisplatin chemoresistance. The *AGPAT3* gene is located on chromosome 21 and has several transcription variants, six of which encode the same protein consisting of 376 amino acids. The specific primers used for expression analysis and overexpression of this gene ensured the detection of all six transcription variants.

At the experimental level, an MTT assay was used to verify the cisplatin resistance/sensitive phenotypes of the A2780cp and A2780 cells, respectively. Accordingly, IC50 values of 62.62 µM and 34.08 µM were determined for the A2780cp and A2780 cells, respectively. Then, RT-qPCR results indicated that the *AGPAT3* gene is upregulated in A2780cp cells compared to the A2780 cells. Furthermore, western blot analysis verified the elevated protein level of AGPAT3 in A2780cp cells compared with A2780 cells. The mTOR signaling pathway has already been reported to be involved in the cisplatin resistance phenotype [31]. Consistently, the mTOR signaling pathway was upregulated in A2780cp cells, as detected at both the mRNA and protein levels. S6K protein is known to act downstream of mTORC1 signaling [32–34]. Interestingly, our western blotting analysis revealed that the phosphorylation of S6K protein was also increased in A2780cp cells. Overall, western blot analysis verified that the p-mTOR/mTOR and p-S6K/S6K ratios are increased in A2780cp cells compared to A2780 cells. These factors might be considered molecular markers of the cisplatin resistance phenotype in A2780cp cells.

Following the successful overexpression of AGPAT3 in A2780 cells, as detected by RT-qPCR and Western blot, the transcription level of mTOR was elevated. The upregulation of mTOR mRNA expression appears to be a consequence of a positive feedback loop involving the mTORC1 target [35], leading to increased stability of mTOR mRNA. Nevertheless, such an effect was not observed at the mTOR protein level. Instead, the ratios of p-mTOR/mTOR and p-S6K/S6K were elevated, suggesting an increased chemoresistance phenotype in the cells.

This effect was further supported by the results of the MTT assay in cells overexpressing AGPAT3. This overexpression resulted in an increased IC50 of A2780 cells to cisplatin, increasing from 31.2 to 38.21 µM. Western blot analysis further confirmed that successful downregulation of AGPAT3 gene expression in A2780cp cells led to drastic reductions in p-mTOR/mTOR and p-S6K/S6K protein levels, reinforcing the regulatory effect of AGPAT3 on the mTORC1 signaling pathway.

Consistently, overexpression of AGPAT3 in A2780 cells resulted in an increased number of live cells and a reduced number of apoptotic cells under cisplatin treatment, as detected by the Annexin PI apoptosis assay. In the absence of cisplatin treatment, this effect was not observed, emphasizing the involvement of AGPAT3 in the drug resistance phenotype.

Furthermore, cell cycle analysis revealed no significant changes in cell cycle phases following AGPAT3 overexpression. However, an increase in the G2/M phase population was observed after AGPAT3 overexpression under cisplatin treatment. An increased G2/M phase population is considered a hallmark of DNA repair system arrest, and it has been reported that mTORC1 causes G2/M arrest [33,34]. Accordingly, we suggest that AGPAT3, through overactivation of mTORC1 signaling, might trigger the DNA repair system and arrest the cell cycle in the G2/M phase under cisplatin treatment. Such alterations in cell cycle distribution may indicate a cellular response to cisplatin-induced DNA damage or stress, allowing for DNA repair before entering mitosis and ensuring the preservation of genomic integrity. Further investigation is required to support this interpretation.

**Fig 7:**
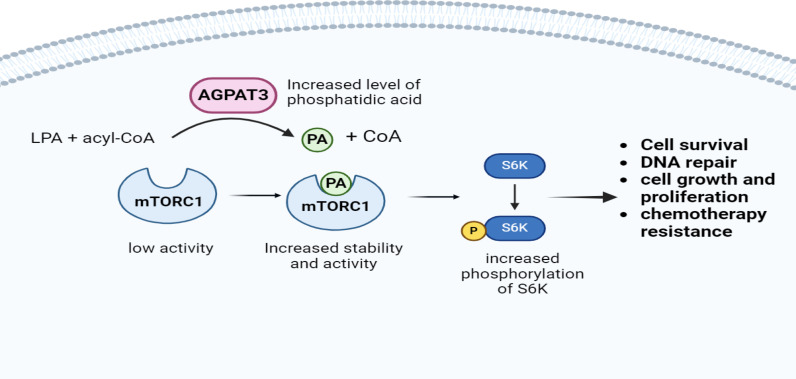
Mechanism of AGPAT3-mediated mTORC1 activation and its downstream effects. This schematic illustrates the role of AGPAT3 in enhancing mTORC1 activity through increased production of phosphatidic acid (PA) and its subsequent effects on S6 kinase (S6K) phosphorylation. AGPAT3 converts lysophosphatidic acid (LPA) and acyl-CoA to PA, leading to elevated PA levels. PA binds to mTORC1, activating the complex. Activated mTORC1 then phosphorylates S6K protein, resulting in increased survival rate, enhanced DNA repair, promotion of cell growth and proliferation, and the development of chemoresistance.

Overall, our findings suggest that AGPAT3 enhances cisplatin resistance in ovarian cancer through mTORC1 induction (Fig 7). Therefore, downregulating AGPAT3 expression or blocking its enzymatic product, phosphatidic acid, from binding to mTOR might be considered therapeutic approaches to overcome cisplatin chemoresistance in ovarian cancer, which has fewer side effects than other classes of mTOR inhibitors do [7]. However, whether AGPAT3 is also involved in other chemoresistance systems and in other cancer types remains to be tested. Therefore, targeting or interfering with enzymes or cofactors that over activate mTORC1 could diminish the side effects of drugs used in cancer therapy. These insights may contribute to the development of more effective treatment strategies in the future.

## Supporting information

S1 Fig Structure of shRNA designed for downregulation of AGPAT3 gene. The structure of the shRNA designed for knocking down of the *AGPAT3* gene. The design was created using IDT OligoAnalyzer website.
(PDF)

S2 Fig Principal Component Analysis (PCA) for RNA-seq data verification. PCA was employed to verify the sample separation in our RNA-seq analysis, specifically to distinguish between resistant and sensitive samples. After performing batch effect removal, the PCA clearly demonstrated the separation of resistant and sensitive samples, indicating distinct expression profiles. Although some samples within the resistant and sensitive groups did not cluster tightly together, the overall PCA results still highlighted significant differences between the resistant and sensitive samples.
(PDF)

S3 Fig Heatmap of differentially expressed genes. The heatmap displays the upregulated and downregulated genes across all samples. It represents the 25 most upregulated genes, including *AGPAT3*, and the 25 most downregulated genes between A2780cp (resistant samples) and A2780 (sensitive samples). *AGPAT3* is consistently upregulated in all resistant samples compared to sensitive samples and ranks 25th based on Log2 fold change (Log2(FC)).
(PDF)

S1 File Differentially expressed genes in A2780 resistant and sensitive to cisplatin ovarian cancer originated cell lines. (XLSX)

S2 File Presenting the original images of Western blotting analysis. (ZIP)

## References

[1] Ferlay J, Ervik M, Lam F. Ovarian cancer incidence and mortalhity. Int Agency Res Cancer GLOBOCAN. 2020:5–6.

[2] De Giorgi U, Casadei C, Bergamini A, Attademo L, Cormio G, Lorusso D, et al. Therapeutic challenges for cisplatin-resistant ovarian germ cell tumors. Cancers (Basel). 2019;11(10):1584. doi: 10.3390/cancers11101584 31627378 PMC6826947

[3] Ranasinghe R, Mathai ML, Zulli A. Cisplatin for cancer therapy and overcoming chemoresistance. Heliyon. 2022;8(9):e10608. doi: 10.1016/j.heliyon.2022.e10608 36158077 PMC9489975

[4] Bowtell DD, Böhm S, Ahmed AA, Aspuria P-J, Bast RC Jr, Beral V, et al. Rethinking ovarian cancer II: reducing mortality from high-grade serous ovarian cancer. Nat Rev Cancer. 2015;15(11):668–79. doi: 10.1038/nrc4019 26493647 PMC4892184

[5] Samuel P, Pink RC, Brooks SA, Carter DR. miRNAs and ovarian cancer: a miRiad of mechanisms to induce cisplatin drug resistance. Expert Review of Anticancer Therapy. 2015;16(1):57–70. doi: 10.1586/14737140.2016.1121107 26567444

[6] Saxton RA, Sabatini DM. mTOR signaling in growth, metabolism, and disease. Cell. 2017;169(2):361–71. doi: 10.1016/j.cell.2017.03.035 28388417

[7] Nguyen T-L, Nokin M-J, Egorov M, Tomé M, Bodineau C, Di Primo C, et al. mTOR inhibition via displacement of phosphatidic acid induces enhanced cytotoxicity specifically in cancer cells. Cancer Res. 2018;78(18):5384–97. doi: 10.1158/0008-5472.CAN-18-0232 30054335

[8] Menon D, Salloum D, Bernfeld E, Gorodetsky E, Akselrod A, Frias MA, et al. Lipid sensing by mTOR complexes via de novo synthesis of phosphatidic acid. J Biol Chem. 2017;292(15):6303–11. doi: 10.1074/jbc.M116.772988 28223357 PMC5391759

[9] Ghosh J, Kobayashi M, Ramdas B, Chatterjee A, Ma P, Mali RS, et al. S6K1 regulates hematopoietic stem cell self-renewal and leukemia maintenance. J Clin Invest. 2016;126(7):2621–5. doi: 10.1172/JCI84565 27294524 PMC4922705

[10] Foster DA, Salloum D, Menon D, Frias MA. Phospholipase D and the maintenance of phosphatidic acid levels for regulation of mammalian target of rapamycin (mTOR). J Biol Chem. 2014;289(33):22583–8. doi: 10.1074/jbc.R114.566091 24990952 PMC4132766

[11] Frias MA, Hatipoglu A, Foster DA. Regulation of mTOR by phosphatidic acid. Trends Endocrinol Metab. 2023;34(3):170–80. doi: 10.1016/j.tem.2023.01.004 36732094 PMC9957947

[12] An D, Zhai D, Wan C, Yang K. The role of lipid metabolism in cancer radioresistance. Clin Transl Oncol. 2023;25(8):2332–49. doi: 10.1007/s12094-023-03134-4 37079212

[13] Li H, Zeng J, Shen K. PI3K/AKT/mTOR signaling pathway as a therapeutic target for ovarian cancer. Arch Gynecol Obstet. 2014;290(6):1067–78. doi: 10.1007/s00404-014-3377-3 25086744

[14] Hsieh H-J, Zhang W, Lin S-H, Yang W-H, Wang J-Z, Shen J, et al. Systems biology approach reveals a link between mTORC1 and G2/M DNA damage checkpoint recovery. Nat Commun. 2018;9(1):3982. doi: 10.1038/s41467-018-05639-x 30266942 PMC6162282

[15] Song L, Liu S, Zhao S. Everolimus (RAD001) combined with programmed death-1 (PD-1) blockade enhances radiosensitivity of cervical cancer and programmed death-ligand 1 (PD-L1) expression by blocking the phosphoinositide 3-kinase (PI3K)/protein kinase B (AKT)/mammalian target of rapamycin (mTOR)/S6 kinase 1 (S6K1) pathway. Bioengineered. 2022;13(4):11240–57. doi: 10.1080/21655979.2022.2064205 35485300 PMC9208494

[16] Holler M, Grottke A, Mueck K, Manes J, Jücker M, Rodemann HP, et al. Dual targeting of Akt and mTORC1 impairs repair of DNA double-strand breaks and increases radiation sensitivity of human tumor cells. PLoS One. 2016;11(5):e0154745. doi: 10.1371/journal.pone.0154745 27137757 PMC4854483

[17] Xie X, Hu H, Tong X, Li L, Liu X, Chen M, et al. The mTOR-S6K pathway links growth signalling to DNA damage response by targeting RNF168. Nat Cell Biol. 2018;20(3):320–31. doi: 10.1038/s41556-017-0033-8 29403037 PMC5826806

[18] El Botty R, Coussy F, Hatem R, Assayag F, Chateau-Joubert S, Servely J-L, et al. Inhibition of mTOR downregulates expression of DNA repair proteins and is highly efficient against BRCA2-mutated breast cancer in combination to PARP inhibition. Oncotarget. 2018;9(51):29587–600. doi: 10.18632/oncotarget.25640 30038706 PMC6049870

[19] Rosas-Plaza X, de Vries G, Meersma GJ, Suurmeijer AJH, Gietema JA, van Vugt MATM, et al. Dual mTORC1/2 inhibition sensitizes testicular cancer models to cisplatin treatment. Mol Cancer Ther. 2020;19(2):590–601. doi: 10.1158/1535-7163.MCT-19-0449 31744897

[20] Lu R, Zhao G, Yang Y, Jiang Z, Cai J, Hu H. Inhibition of CD133 overcomes cisplatin resistance through inhibiting PI3K/AKT/mTOR signaling pathway and autophagy in CD133-positive gastric cancer cells. Technol Cancer Res Treat. 2019;18:1533033819864311. doi: 10.1177/1533033819864311 31405336 PMC6693020

[21] Zhu H, Wu J, Zhang W. PKM2 enhances chemosensitivity to cisplatin through interaction with the mTOR pathway in cervical cancer. Scientific Reports. 2016;6. 10.1038/srep30788 PMC4974606 27492148

[22] Xu W, Wei Y, Li Y, Yin Y, Yuan W, Yang Y, et al. TAZ inhibition restores sensitivity of cisplatin via AKT/mTOR signaling in lung adenocarcinoma. Oncol Rep. 2017;38(3):1815–21. doi: 10.3892/or.2017.5847 28737828

[23] Song L, Duan P, Gan Y, Li P, Zhao C, Xu J, et al. Silencing LPAATβ inhibits tumor growth of cisplatin-resistant human osteosarcoma in vivo and in vitro. Int J Oncol. 2017;50(2):535–44. doi: 10.3892/ijo.2016.3820 28035350

[24] Ruibin J, Guoping C, Zhiguo Z, Maowei N, Danying W, Jianguo F, et al. Establishment and characterization of a highly metastatic ovarian cancer cell line. Biomed Res Int. 2018;2018:3972534. doi: 10.1155/2018/3972534 30046596 PMC6036838

[25] Tudrej P, Kujawa KA, Cortez AJ, Lisowska KM. Characteristics of in vivo model systems for ovarian cancer studies. Diagnostics (Basel). 2019;9(3):120. doi: 10.3390/diagnostics9030120 31540126 PMC6787695

[26] Douvas MG, Hogan KN, Ji Y, Hollenback D, Bonham L, Singer JW, et al. Effect of lysophosphatidic acid acyltransferase-beta inhibition in acute leukemia. Leuk Res. 2006;30(8):1027–36. doi: 10.1016/j.leukres.2005.11.018 16488473

[27] Blaskovich MA, Yendluri V, Lawrence HR, Lawrence NJ, Sebti SM, Springett GM. Lysophosphatidic acid acyltransferase beta regulates mTOR signaling. PLoS One. 2013;8(10):e78632. doi: 10.1371/journal.pone.0078632 24205284 PMC3814986

[28] Niesporek S, Denkert C, Weichert W, Köbel M, Noske A, Sehouli J, et al. Expression of lysophosphatidic acid acyltransferase beta (LPAAT-beta) in ovarian carcinoma: correlation with tumour grading and prognosis. Br J Cancer. 2005;92(9):1729–36. doi: 10.1038/sj.bjc.6602528 15841084 PMC2362024

[29] Springett GM, Bonham L, Hummer A, Linkov I, Misra D, Ma C, et al. Lysophosphatidic acid acyltransferase-beta is a prognostic marker and therapeutic target in gynecologic malignancies. Cancer Res. 2005;65(20):9415–25. doi: 10.1158/0008-5472.CAN-05-0516 16230405

[30] Diefenbach CSM, Soslow RA, Iasonos A, Linkov I, Hedvat C, Bonham L, et al. Lysophosphatidic acid acyltransferase-beta (LPAAT-beta) is highly expressed in advanced ovarian cancer and is associated with aggressive histology and poor survival. Cancer. 2006;107(7):1511–9. doi: 10.1002/cncr.22184 16944535

[31] Pan Z, Zhang H, Dokudovskaya S. The role of mTORC1 pathway and autophagy in resistance to platinum-based chemotherapeutics. Int J Mol Sci. 2023;24(13):10651. doi: 10.3390/ijms241310651 37445831 PMC10341996

[32] Yang L, Miao L, Liang F, Huang H, Teng X, Li S, et al. The mTORC1 effectors S6K1 and 4E-BP play different roles in CNS axon regeneration. Nat Commun. 2014;5:5416. doi: 10.1038/ncomms6416 25382660 PMC4228696

[33] Wu X, Xie W, Xie W, Wei W, Guo J. Beyond controlling cell size: functional analyses of S6K in tumorigenesis. Cell Death Dis. 2022;13(7):646. doi: 10.1038/s41419-022-05081-4 35879299 PMC9314331

[34] Ghosh J, Kapur R. Role of mTORC1-S6K1 signaling pathway in regulation of hematopoietic stem cell and acute myeloid leukemia. Exp Hematol. 2017;50:13–21. doi: 10.1016/j.exphem.2017.02.004 28342808 PMC5569945

[35] Mura M, Hopkins TG, Michael T, Abd-Latip N, Weir J, Aboagye E, et al. LARP1 post-transcriptionally regulates mTOR and contributes to cancer progression. Oncogene. 2015;34(39):5025–36. doi: 10.1038/onc.2014.428 25531318 PMC4430325

