# Supplementary figures and images for: Introduction of AGPAT3 gene as a regulator of cisplatin resistance in A2780 ovarian endometrioid carcinoma cell line
Source: PLoS One. 2025 Mar 10;20(3):e0318740. doi: 10.1371/journal.pone.0318740 (PMC11892817; doi:10.1371/journal.pone.0318740)

### Sup. Figure 3

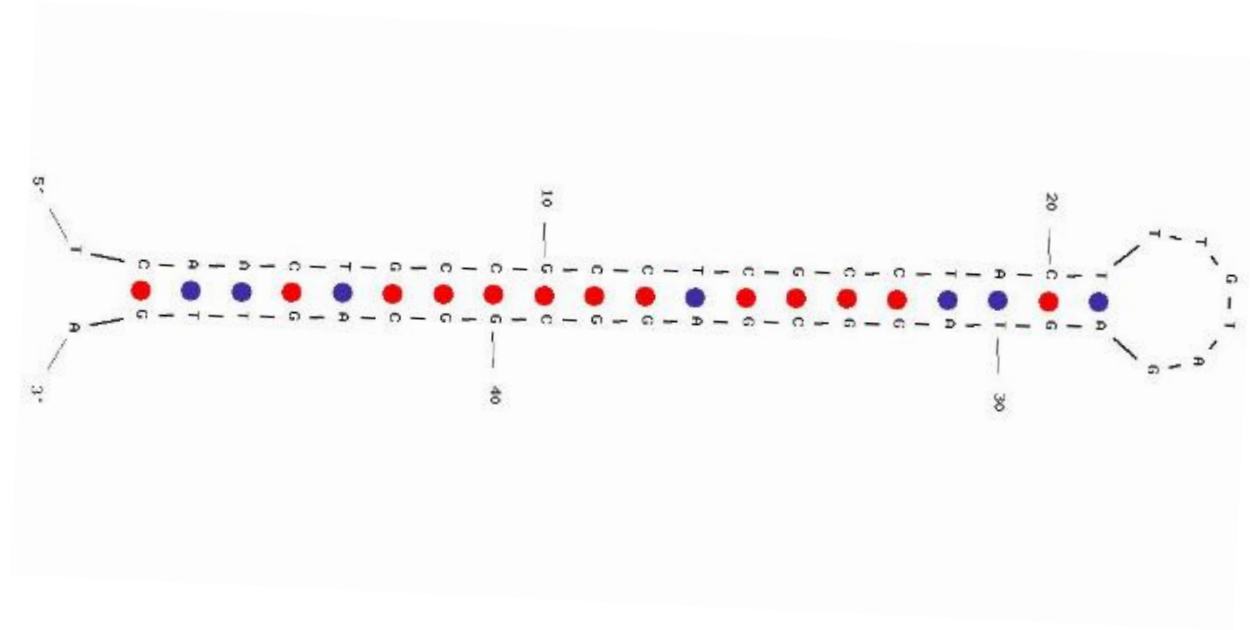

Supplement: S1 Fig — The structure of the shRNA designed for knocking down of the AGPAT3 gene. The design was created using IDT OligoAnalyzer website. (PDF) [file pone.0318740.s001.pdf]

Sup. Figure 2

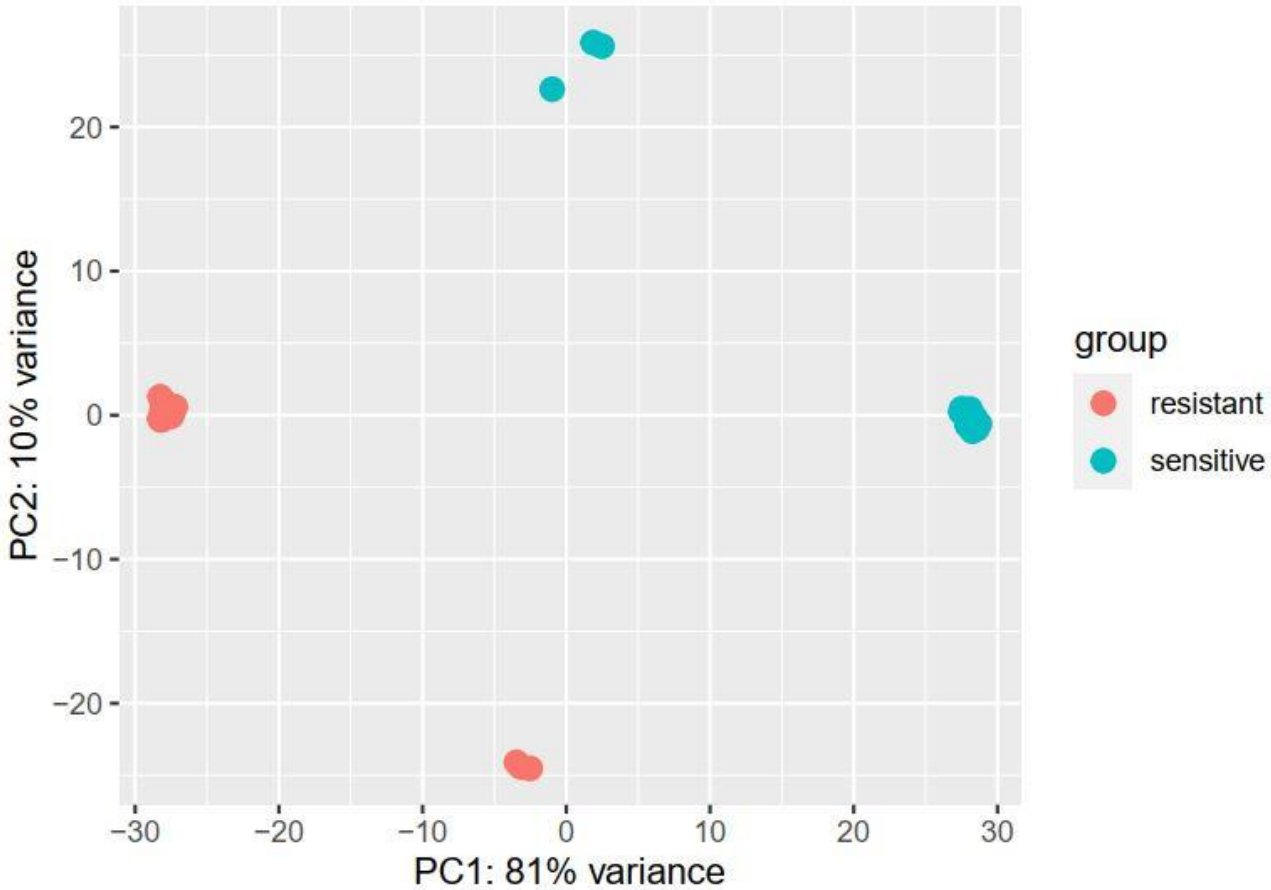

21 A2780cp and 21 A2780 samples

Supplement: S2 Fig — PCA was employed to verify the sample separation in our RNA-seq analysis, specifically to distinguish between resistant and sensitive samples. After performing batch effect removal, the PCA clearly demonstrated the separation of resistant and sensitive samples, indicating distinct expression profiles. Although some samples within the resistant and sensitive groups did not cluster tightly together, the overall PCA results still highlighted significant differences between the resistant and sensitive samples. (PDF) [file pone.0318740.s002.pdf]

Sup. Figure 1

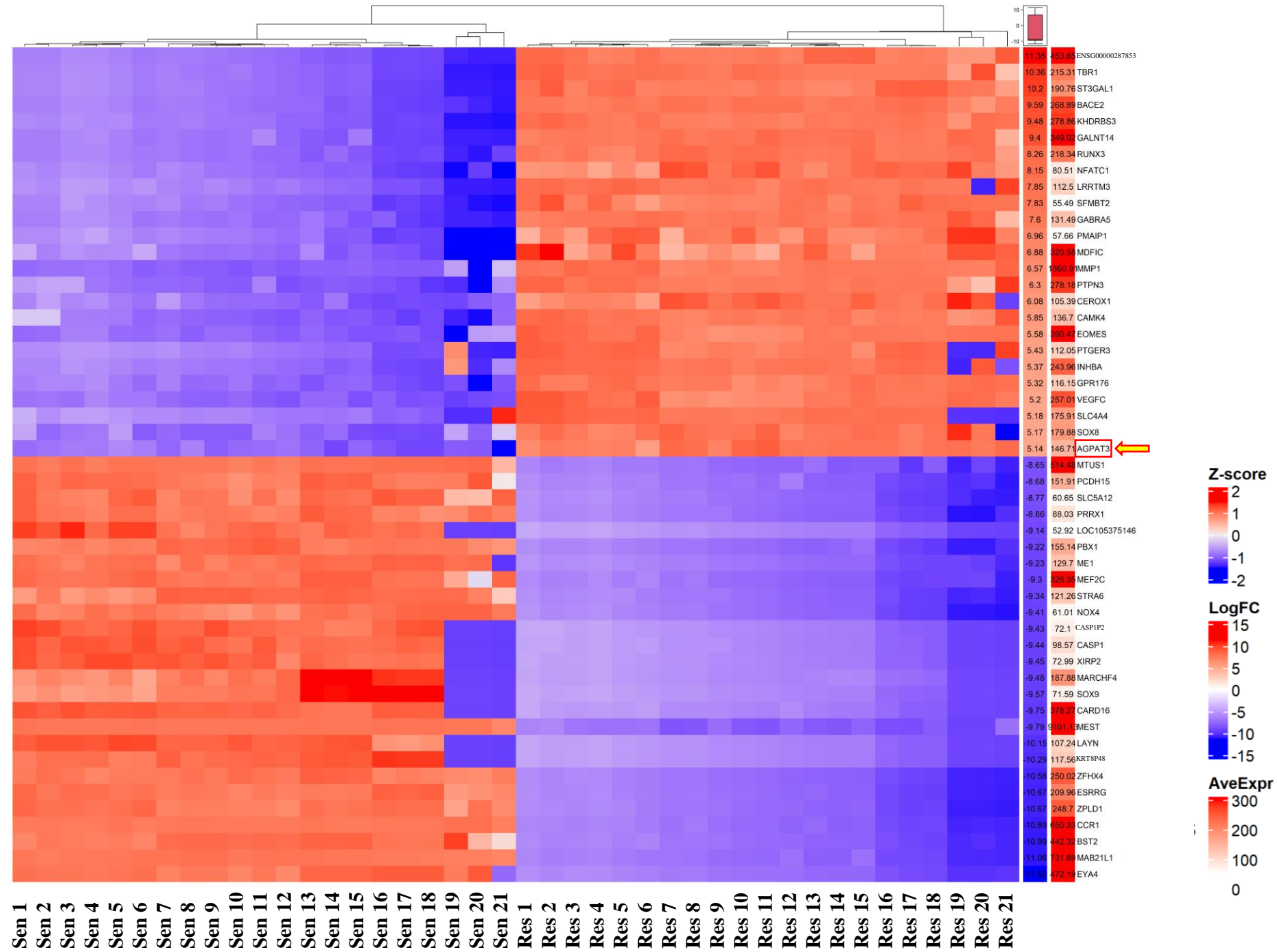

Supplement: S3 Fig — The heatmap displays the upregulated and downregulated genes across all samples. It represents the 25 most upregulated genes, including AGPAT3, and the 25 most downregulated genes between A2780cp (resistant samples) and A2780 (sensitive samples). AGPAT3 is consistently upregulated in all resistant samples compared to sensitive samples and ranks 25th based on Log2 fold change (Log2(FC)). (PDF) [file pone.0318740.s003.pdf]

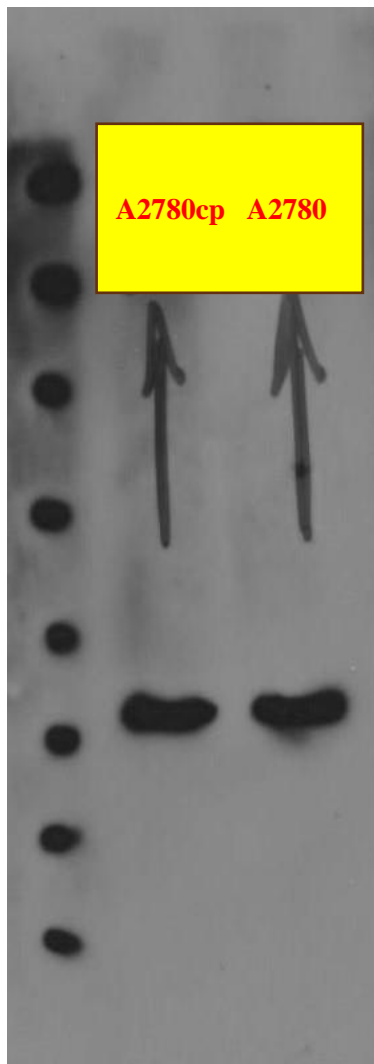

**B-act**

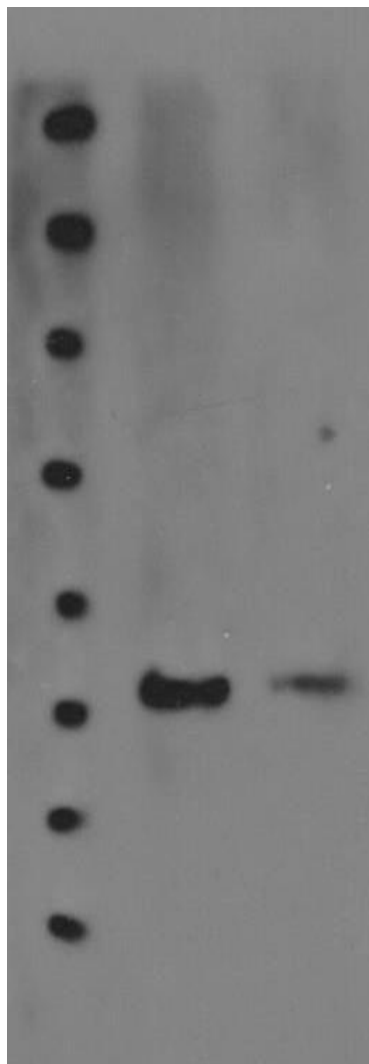

**AGPAT**

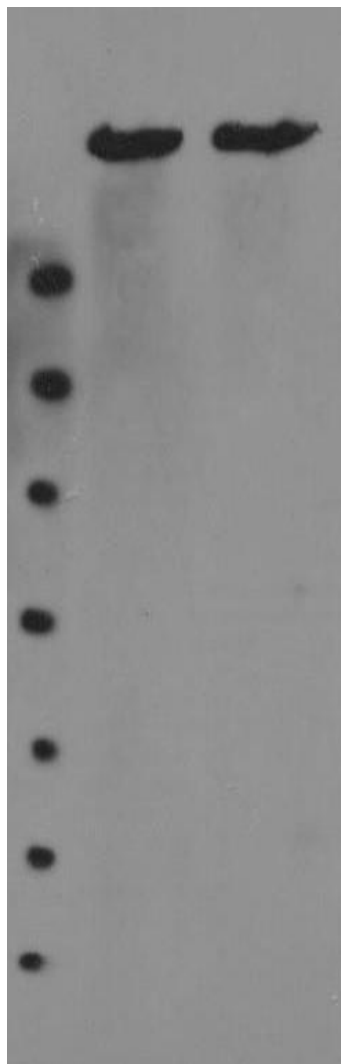

**mTOR**

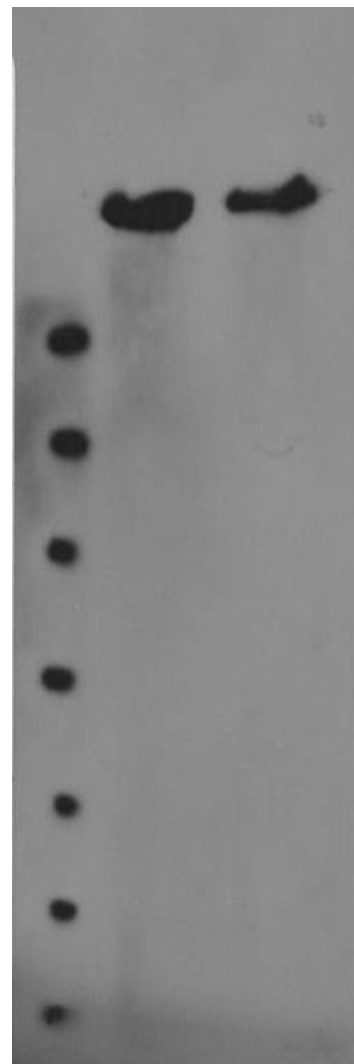

**p-mTOR**

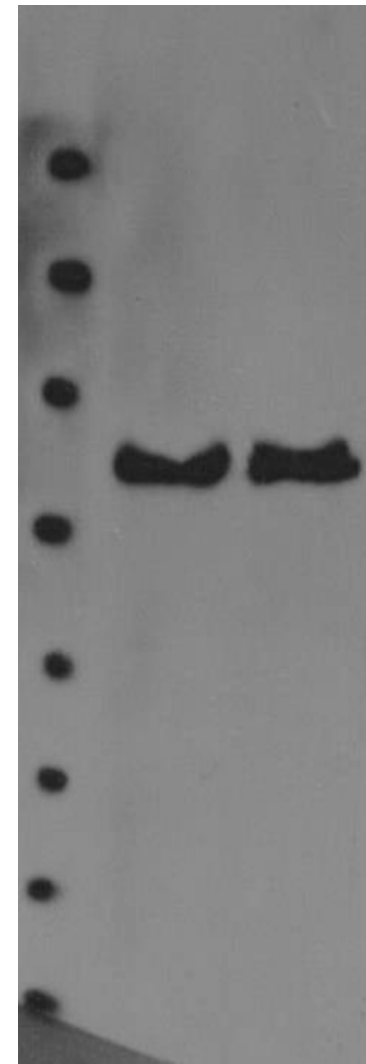

**S6K**

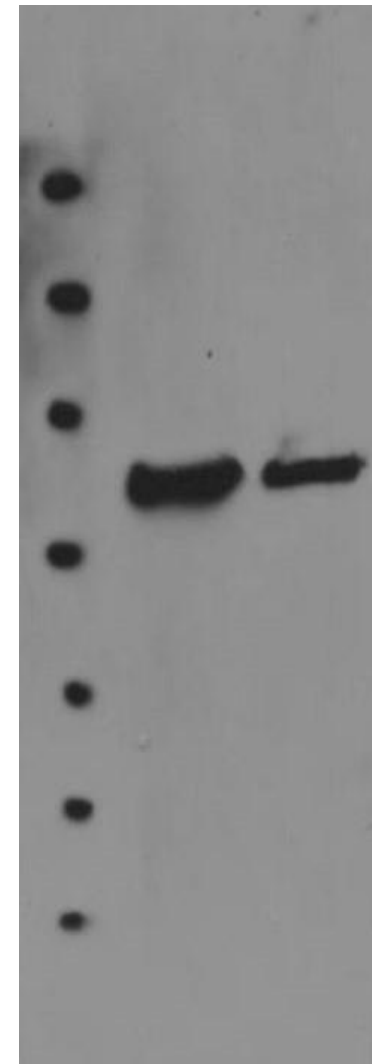

**p-S6K**

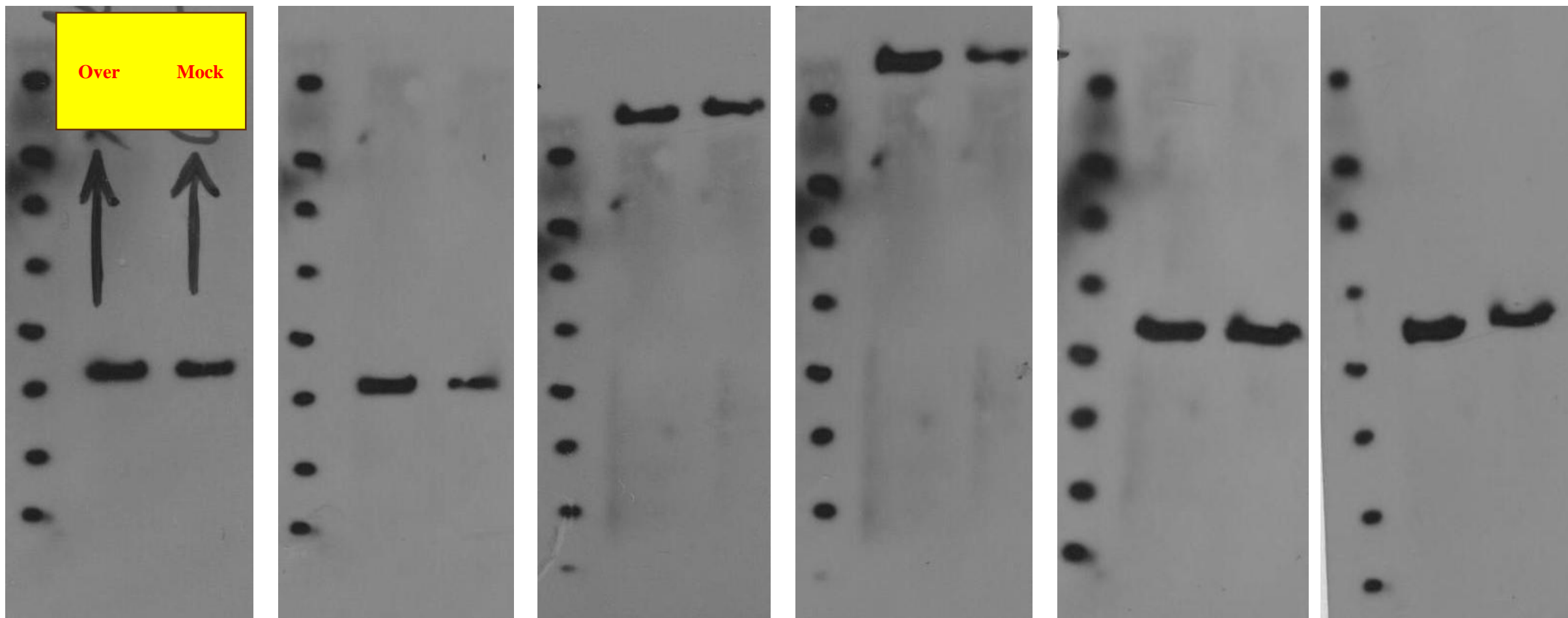

**B-act**

**AGPAT**

**mTOR**

**p-mTOR**

**S6K**

**p-S6K**

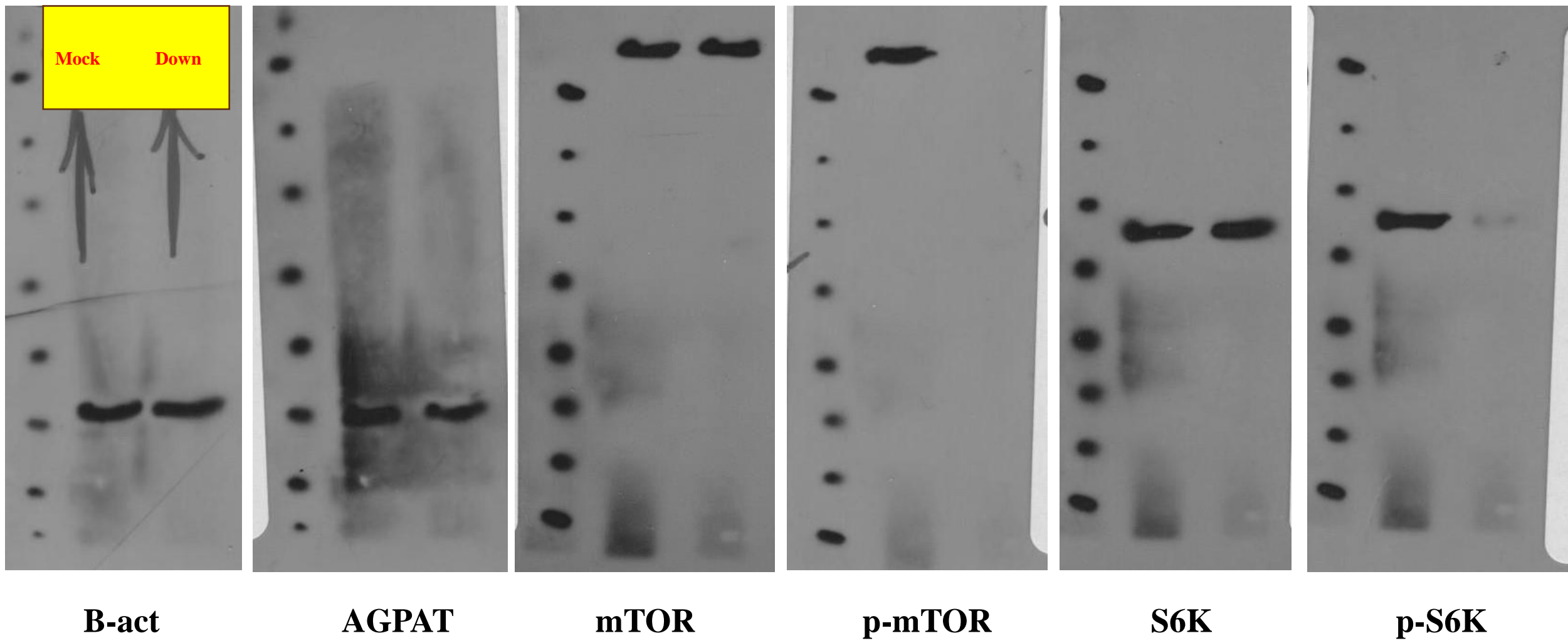

Western blot ladder

Thermo SCIENTIFIC  
84785

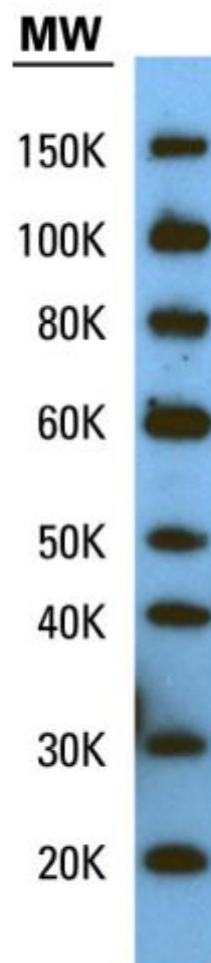

Supplement: S2 File — (ZIP) [file pone.0318740.s005.zip › Supplementry (Western blot)-merged.pdf]
